# Exploring a long short-term memory for mountain flood forecasting based on watershed-internal knowledge graph and large language model

**DOI:** 10.1371/journal.pone.0318644

**Published:** 2025-03-13

**Authors:** Songsong Wang, Ouguan Xu

**Affiliations:** 1 School of Computer Science and Technology, Zhejiang University of Water Resources and Electric Power, Hangzhou, China; 2 College of Engineering, Ocean University of China, Qingdao, China; University 20 Aout 1955 skikda, Algeria, ALGERIA

## Abstract

The water levels associated with mountain floods exhibit rapid fluctuations within small watersheds, necessitating extensive data on various factors influencing such disasters to facilitate real-time forecasting. This study investigates the application of Long Short-Term Memory (LSTM) networks for mountain flood forecasting, designing a watershed-internal Knowledge Graph (KG) and Large Language Model (LLM) that encompass watershed relationships and internal information structures. We have developed a hydrological KG for the Qixi Reservoir and Qiaodongcun forecasting points located in Zhejiang Province, China, to systematically organize water conservancy data, identify significant disaster-related factors, optimize the input hydrological data, and determine the most effective combination of input data for forecasting water levels. Additionally, we have implemented Recurrent Neural Networks (RNN) and Gated Recurrent Units (GRU) for comparative analysis with LSTM. The findings indicate that the LSTM model, when integrated with the watershed-internal KG and LLM, can effectively incorporate critical elements influencing water level changes, the accuracy of the LLM-KG-LSTM model is enhanced by 3% compared to the standard LSTM model, and the LSTM series outperforms both RNN and GRU models, Our method will guide future research from the perspective of focusing on forecasting algorithms to the perspective of focusing on the relationship between multi-dimensional disaster data and algorithm parallelism.

## 1 Introduction

The water level’s change is rapidly, and requires high accuracy and real-time performance in small watersheds’ mountain floods forecasting. There are many points of water level monitoring and forecasting, and the large number of disasters causing factors. Real-time information is related to hydrology (water level, rainfall, etc.) [1], meteorology (weather status, future rainfall, temperature, humidity, wind speed, wind direction, etc.), geography [2], and social profiles. How to systematically manage water conservancy Big Data (BD) in small watersheds [3], has become the main issue determining the large-scale data-driven water level forecasting in small watersheds. Accurate real-time prediction is required for short-term 24-hour and mid-term 1 ~ 7 days water levels. The small watershed of mountain floods changes rapidly and its predictability is not as regular as that of large watersheds, making data mining very important.

Large-scale data collection, processing, and storage have been employed to develop a unified methodology for forecasting water levels at numerous forecasting points [4]. Traditionally, individual monitoring stations are the primary focus. However, there is now a necessity to establish integrated solutions suitable for broad application across smaller watersheds. The sustainability of time series data demands consistent collection, rational processing, and efficient storage [5]. Although a lot of efforts have been made in the prediction algorithm, with the model becoming more and more large-scale and faster and faster, the prediction accuracy has not been improved. The key is the lack of effective organization of sample data, reasonable preprocessing and typical feature extraction. In general, the quality of prediction depends not only on the high performance of the model, but also on the importance of data. Solving problems from the perspective of data organization, especially the prediction of mountain torrents, has practical significance for reducing the risk of mountain torrents. In addition, it will provide new ideas for the bottleneck prediction of the model. Based on the accuracy and real-time, this paper will have certain advantages for the real-time and reliable prediction of mountain torrents in complex water network.

In this paper, we try to adopt a real-time data integration processing method to more efficiently utilize data features the Qixi Reservoir forecasting point of Qiantang River watershed, and Qiaodongcun forecasting point of Tiaoxi watershed by extreme water level change, Zhejiang Province of China. We use real-time water conservancy BD to achieve forecasting of mountain flood water levels, including real time water conservancy BD acquisition, organization, storage and a series of data processing methods. We introduce hydrological Knowledge Graph (KG) and Large Language Model (LLM) to organize water conservancy data, fully explore the strongly related disaster causing factors of mountain floods, optimize the input sample hydrological data, and achieve the optimal combination of input data for water level forecasting points. We build the LSTM time forecasting model, and store a large amount of real-time data for the convenience of training as samples for the next forecasting. Based on the hydrology KG, an efficient forecasting structure is designed to achieve real-time forecasting of water levels at multiple points of small watershed mountain flood.

## 2 Related works

With the development of intelligent water conservancy and Internet of Things (IoT), real-time data processing and storage, this integrated processing method is possible[6]. In the intelligent IoT method for water conservancy, interconnection and interoperability operation technology can achieve real-time data collection, cooperate with enrich data from the perspective of multi-source data. In terms of data organization, KG can express and clarify the relationships between various data forecasting points and data. In terms of data storage, design an efficient water conservancy database to improve storage efficiency, reduce storage space, and ensure data quality [7]. Nowadays, the scale of KG is becoming larger and larger, and the traditional graph construction and completion are also facing many difficulties, such as data acquisition, entity recognition, knowledge extraction and entity disambiguation. The construction of large-scale KG often requires a lot of manpower, material resources and time costs, and still cannot guarantee the quality and availability of KG. The LLM can effectively solve these problems [8,9]. There is a large amount of knowledge information in the LLM. When dealing with complex text data information, it can quickly carry out entity recognition and extraction, and effectively respond to the challenge of knowledge construction and completion. In addition, link forecasting is the key step of KG reasoning and question answering. In zero sample and small sample learning, LLM can also effectively mine the logical relationship between entities.

In terms of the feature engineering, the forecasting of mountain floods requires the support of BD, especially in the case of large-scale forecasting points in small watersheds [10]. In data-driven forecasting methods, data feature engineering is crucial [11]. Feature engineering is the key to exhibit optimal (or near optimal) performance on unknown data[12]. From a mathematical perspective, feature engineering is the process of manually designing input variable. Mainly for semantic, relationship and time consistency between data, data encoding, normalization and standardization. To ensure the reliability of data application in DL forecasting from the source of data, it is necessary to go through the entire process of data acquisition, organization, storage, and retrieval by using the IoT [13].

The more obvious the characteristics of mountain floods induced disaster data, the less complex forecasting models would be selected, while also running faster and easier to maintain [14]. Good features can achieve good performance even when the parameters are not optimal, reducing the workload and time of parameter tuning, and greatly reducing model complexity. The purpose of feature engineering is originally to improve the performance of the models[15].

In terms of data organization structure, the effective way to organize BD in water conservancy is through KG [16]. The KG mainly includes key processes such as knowledge representation, extraction, fusion, inference, and storage [17]. It aims to use graph models to describe the concepts and internal relationships between entities that exist in the real world [18]. The triple structure of “entities, knowledge, and relationships” is suitable for expressing the complex network relationships between watersheds, river networks, etc. In addition, with the increasing demand for metadata in water conservancy informatization and intelligence, it is necessary to improve the completeness of data and organize the structural relationships between data in order to achieve dynamic information exchange and processing for data retrieval [19]. For example, dynamic and static data such as watershed flood control plans, scheduling plans for typical water and drought disaster events, expert experience, real-time hydrological data, and equipment data can support rapid decision-making [20]. Excellent data organization enhances the utilization value of data.

Zhejiang province of China has many small watersheds and abundant water conservancy information resources, which provide a water conservancy digitization foundation for constructing small watersheds based on dynamic KG [21]. This paper will be based on the small watersheds in Zhejiang Province, study the dynamic water conservancy KG model, design a small watersheds flood disaster KG system, implement a knowledge inference method based on inference rules, apply it to the construction and retrieval system of water conservancy information KG [22], and carry out visualization of small watersheds dynamic KG, water level forecasting, and real-time monitoring of reservoirs to support large-scale forecasting points [23].

In terms of construction KG, Large Language Model (LLM) and KG began to integrate with each other, LLM-augmented KG [24]. Due to their strong organizational capabilities, LLMs are considered as a possible alternative to structured knowledge [25]. At the same time, incorporating LLM in KG is regarded as a promising approach for enhancing knowledge reasoning [26]. The LLM after KG is adopted is more accurate in answering questions, and LLM makes the knowledge acquired based on KG more comprehensive.

KG and Large LLM are both the representations of knowledge. KG is a symbolic knowledge base with certain reasoning ability, and the results can be explained well. But there are some shortcomings, such as high construction cost, lack of generalization ability, and difficult to update. LLM is a parameterized probabilistic knowledge base with strong semantic understanding and generalization ability, but it is a black box model, which may fabricate false content, and the interpretability of the results is poor. It can be seen that using LLM and KG together and taking advantage of their advantages is a complementary approach [8,27].

In terms of mountain flood level forecasting model, by using Deep Learning (DL) series forecasting to verify the real-time reliability of integrated processing methods, the DL forecasting method including Recurrent Neural Network (RNN), Gated Recurrent Units (GRU) and Long Short-Term Memory (LSTM), it is easy to reflect the effect by selecting different feature values. RNNs has been used for sequence modeling and has achieved good results in various natural language processing tasks [28]. At its core, the RNN network can learn all the hidden states within the time series before forecasting as feature representations of past information, and combine the current input to provide the next forecasting result [29,30]. At each time step, new observations can be used to continuously recursively update the hidden state. Therefore, the RNN was also the earliest applied in time forecasting scenarios in DL methods. Early RNN variants may be limited by gradient explosion and vanishing problems when learning long-term serial dependencies in data.

In recent years, various improved methods of the DL series have been widely applied to flood forecasting at specific locations[31,32]. One solution is to use Long Short-Term Memory (LSTM) network [33]. The design inspiration for long-term and short-term memory networks comes from the logic control gates of computers by using various gates to control memory elements, such as by using output gates to control the output sequence from the unit [34], by using input gates to determine when to read data into the unit, by using forget gates to manage the content of reset units. The weights obtained through training in LSTM can determine when to remember or ignore inputs in hidden states. Therefore, LSTM and its variant Gated Recurrent Units (GRU) units have become important components of RNN based time series forecasting. Further improvements are data-driven, integrated hydrological modelling[35,36].

Relevant variants of LSTM method and other methods are also the main research direction. The hybrid model [37] is a major trend, for example, incorporating hydraulic constraints with DL [38] can improve the interpretability of the model. LSTM outperformed boosted regression tree with a significant difference of accuracy [39]. Radial basis function neural network [40,41] and using Kalman filtering [42] can effectively improve the forecasting effect of single DL. In addition, some studies began to pay attention to the importance of data. Multi source and multi-dimensional data are used for forecasting, which has certain advantages [43]. A multi-source data driven model has been widely used [44]. The relevant DL prediction methods have achieved good forecasting results, but these methods focus on how to mine the rules of fixed data sets, and lack of thinking about the rational combination of data.

Overall, from data generation to application, integration of KG and LLM methods can improve data utilization, especially in terms of timeliness. At present, they have a good foundation in their respective dispersion methods. By integrating resources and constructing a data processing system, real-time, accurate, and reliable data sources are provided for dynamic and real-time mountain flood forecasting, which is suitable for forecasting large-scale water level forecasting points.

## 3 Integrated processing structure by KG and LLM

### 3.1 Building the watershed-internal KG

**Fig 1:**
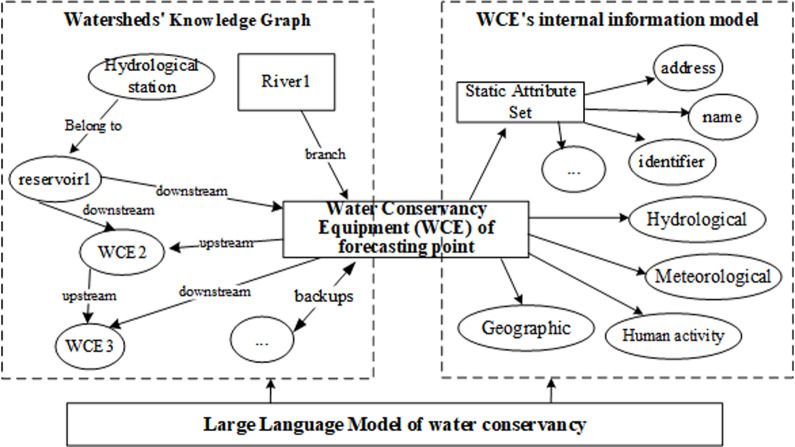
The struct of small watersheds’ external relationship KG and entity of WCE’s internal information KG by LLM.

We build a watershed-internal KG of Water Conservancy Equipment (WCE) information of forecasting point, and form a water conservancy KG through information extraction, data fusion, and knowledge processing. according to the requirements and general specifications of the semantic interoperability data dictionary, the attribute element structure of WCE is constructed in the attributes of the bottom layer of WCE of KG, as shown in Fig 1. The struct of watershed-internal KG includes watersheds’ external relationship and entity of WCE’s internal information by LLM.

Through water conservancy LLM, the WCE adjacent to the forecasting point is identified, and the relevant hydrology, meteorology, geography, human activities, etc. are given to build a professional water conservancy KG model. In addition, the LLM realizes the automatic identification of workflow and multi-business data association on the basis of semantic understanding, and endows the reasoning ability of water conservancy business process of water conservancy KG. We use the ontology construction method of multi service fusion KG to digitally describe the water conservancy objects and elements in the basin environment from multi-temporal and multi-scale, forming a digital water conservancy business logic flow. The LLM aid knowledge mining is applied to realize the association of objects, models and data among multiple businesses, and complete the construction of KG based on the LLM.

Knowledge reasoning aims to infer new facts based on existing KG. This study conducts knowledge inference based on inference rules, mining the knowledge of the Qiantang River Basin hidden in the Qiantang River watershed KG. Firstly, based on the existing information KG of the Qiantang River watershed, combined with knowledge in the field of water conservancy, define inference rules. Table 1 shows some inference rules and their explanations. For example, in the conceptual layer of the graph, there is an inflow relationship between river concepts and reservoir concepts, a belonging relationship between reservoir concepts and hydrological station concepts, while there is no relationship between river concepts and hydrological station concepts. However, based on domain knowledge, it is known that there is a positional relationship between hydrological stations and rivers. Therefore, inference rule 1 can be defined to obtain the river where the hydrological station is located through the reservoir. Based on the relationship, relevant river basins, upstream and downstream relationships can be found to quickly combine relevant input data.

**Table 1:** Inference rules and explanations of the watershed-internal KG.

ID	Inference rules	Explain
1	(River, membership, reservoir), (hydrological station, belonging to, reservoir) → (hydrological station, located in, river)	The hydrological station is a monitoring station for the reservoir on the river to which it belongs
2	(pump station/sluice, including water intake), (pump station/gate station, belonging to, lake), (lake, belonging to, watershed partition) → (Water intake, belonging to, watershed partition)	The water intake belongs to the watershed division of the lake where the pumping station/gate station that owns the water intake is located
3	(Hydrological station, upstream, (sluice, belonging to, river) → (Hydrological station, upstream, sluice)	The hydrological station is located upstream of the sluice on the river

Through KG, it is convenient to organize multidimensional data in the water conservancy industry to achieve better data results. KG also play an important role in BD analysis. With the continuous growth of BD, analyzing and understanding the complexity and diversity of this data has become increasingly difficult. KG, as a way to express knowledge and semantic associations, can help us understand and analyze data more deeply.

### 3.2 Integrated process for water conservancy BD

**Fig 2:**
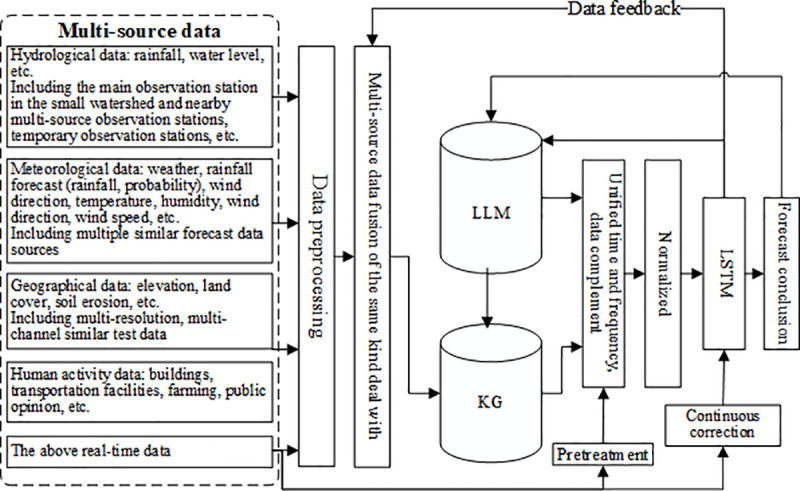
Overall scheme of multi-source heterogeneous data processing.

The integrated data platform connects to the multi-source data of the main influencing factor, such as hydrology, meteorology, geography, human activities, and combine with the R&D team’s water conservancy database to build a data center to achieve large-scale collection of historical, real-time and forecast data. After data preprocessing, data fusion, storage and other processes for the compound forecasting model to use, and model parameters into the model database, the overall plan of multi-source data processing is shown in Fig 2.

The water conservancy BD processing module includes data digitization, data completion, unifying time-frequency of each information based on hourly resolution, revising, normalizing, standardizing, and segmenting data. Supplementing missing data through data sequence mean and replacing character class enumeration (wind direction data) with numerical numbering; Verify and revise the hydrological data of the forecasting point by using historical meteorological and predicted weather data from the county and city where the forecasting point is located; And unify hydrological, meteorological and other data by hour; Remove abnormal data and ensure that all field data is within a reasonable range; Maintain a unified unit for data of the same type and field, and use normalization and standardization methods to remove units from the data. The data of each field is limited to the range of [0,1]; Continuously segment the data and provide a unified JSON data package as needed.

In addition, large-scale instruments such as terrain scanning drone terrain collectors, multi-wave speed river terrain survey boats and other large instruments are used to collect high-density geographic data in the test area; a water conservancy data mobile IoT device is built at the test site to collect real-time hydrometeorological and geographic data to improve real-time and accuracy of model input data.

### 3.3 Forecasting model based on LSTM

**Fig 3:**
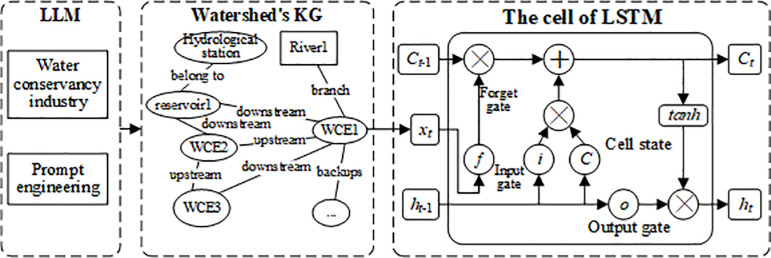
The forecasting model based on LSTM with LLM and watershed’s KG.

The structure of the improved LSTM by LLM and KG is shown in Fig 3. We design three progressive models, such as LSTM, KG-LSTM and LLM- KG-LSTM model. Based on the cell of LSTM, by constructing the KG of the basin, it can be quickly organized in the case of multiple forecasting points, including hydrological stations, reservoirs and other WCEs. In addition, the KG of watershed can be constructed more quickly and perfectly through the water conservancy knowledge and prompt words of LLM.

The gates and states’ equations of LSTM are in the Table 2, and the parameter’s interpretation are in the Table 3. The rationale behind this structural design is that the cell state serves as a memory unit, retaining valuable information via the distinct operations of each gate. The input gate, with the help of current inputs and historical data, determines what new information to store in the cell. Conversely, the forget gate enables the cell to remove certain old information, effectively clearing its content. Finally, the output gate controls the selective release of information.

**Table 2:** LSTM model’s equations.

Name	Equation
Input gate	$i_{t}=\sigma \left(W_{i}x_{t}+U_{i}h_{t-1}+b_{i}\right)$
Forget gate	$f_{t}=\sigma \left(W_{f}x_{t}+U_{f}h_{t-1}+b_{f}\right)$
Output gate	$O_{t}=\sigma \left(W_{O}x_{t}+U_{O}h_{t-1}+b_{O}\right)$
Cell state of input node	$\tilde{C_{t}}=\text{tanh}\left(W_{c}x_{t}+U_{c}h_{t-1}+b_{c}\right)$
Cell state	$C_{t}=f_{t}⊗C_{t-1}+i_{t}⊗\tilde{C_{t}}$
Hidden state	$h_{t}=O_{t}⊗\text{tanh}\left(C_{t}\right)$

**Table 3:** Parameter’s interpretation.

Param	Interpretation
$x_{t}$	The input data, including hydrological, meteorological, geographic, and crowdsourced data on the same time scale.
*i*_*t*_	The input gate
*f*_*t*_	The forget gate
*o*_*t*_	The output gate
*W*_*i*_	The weight of connecting input data and the input gate
*W*_*f*_	The weight of connecting input data and the forget gate
*W*_*o*_	The weight of connecting input data and the output gate
*U*_*i*_	The weight of connecting input gate and the hidden state
*U*_*f*_	The weight of connecting forget gate and the hidden state
*U*_*o*_	The weight of connecting output gate and the hidden state
*b*_*i*_	The input gate bias vector
*b*_*f*_	The Forget gate bias vector
*b*_*o*_	The output gate bias vector
*C*_*t-*1_	The cell input
*C*_*t*_	The current cell state
*h*_*t*_	The current hidden state
*σ*	The logistic sigmoidal function
⊗	The element-wise multiplication (dot product)

## 4 Experiment and results

### 4.1 Data

The Qiantang River watershed is mountainous area, and the Qixi Reservoir forecasting point is in the Kaihua County, Zhejiang Province of China, which is the source of the Qiantang River. The climate in Kaihua County belongs to the subtropical (northern edge) monsoon climate, characterized by warmth, humidity, abundant rainfall, and distinct four seasons. The annual average precipitation is 1990 mm. The Tiaoxi watershed is located in the southern part of Zhejiang Province in Eastern China, which is the typical small watersheds and mountain flood prone. The main mountain range in the area is Tianmu Mountain. Qiaodongcun forecasting point is in the Lin’an District, Tiaoxi watershed, Zhejiang Province of China, the water level changes rapidly, and extreme water levels are often difficult to predict.

The inputs connect to the multi-source data of the main influencing factor data platform such as hydrology, meteorology, geography, human activities, and combine with the R&D team’s water conservancy database to build a data center to achieve large-scale collection of historical, real-time and forecast data. After data preprocessing, data fusion, storage and other processes for the DL forecasting model to use, and model parameters into the model database. The main data are shown in Table 4. Because about 20% of the missing errors in the process of sensor transmission, the missing data is supplemented by linear or by reference to the adjacent forecasting points, which is also the advantage of watershed-internal KG to find the adjacent points.

**Table 4:** Collection of data on the impact factors of mountain floods.

Type	Impact factors	Characteristics	Multimodal	Sources
Hydrology	rainfall, water level, etc.	Including the main observation station in the small watershed and nearby multi-source observation stations, temporary observation stations, etc. The data changes rapidly, counting every hour to improve accuracy through redundancy.	digit, image (water gauge)	Historical data: the government department, https://sqfb.slt.zj.gov.cn/. Real time data: water conservancy data Internet of Things device is built at the test site.
Meteorology	weather, rainfall forecast (rainfall, probability), wind direction, temperature, humidity, wind direction, wind speed, etc.	Including multiple similar forecast data sources. The data changes rapidly, counting every hour to improve accuracy through redundancy.	text, digit, image	https://data.cma.cn
Geography	elevation, land cover, soil erosion, etc.	Including multi-resolution, multi-channel similar data. The data has less variation and is related to the test points.	image	The government department, Internet, and terrain scanning drone terrain collectors, multi-wave speed river terrain survey boats and other large instruments are used to collect high-density geographic data in the test area.
Human activities	buildings, transportation facilities, farming, public opinion, etc.	Has a certain degree of contingency.	Image, map, digit	The government department, Internet.

**Fig 4:**
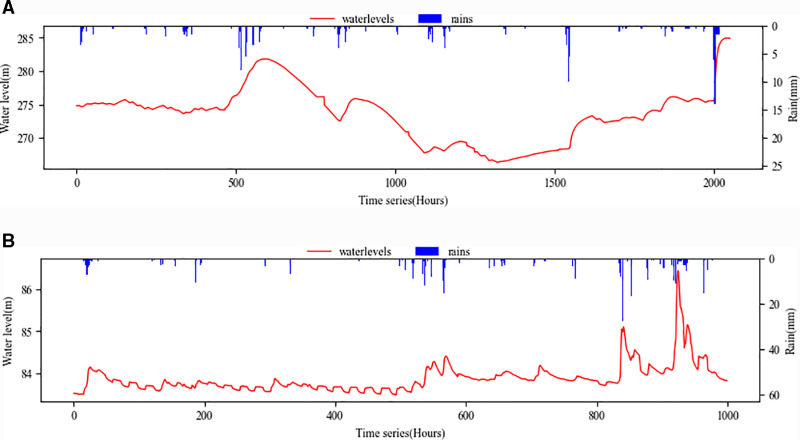
Rainfall and water level data. (a) Qixi Reservoir’s forecasting points from February 25th, 2021 to May 25th, 2021. (b) Qiaodongcun’s forecasting points from May 29, 2020 to July 9, 2020.

The multi-source data of Qixi reservoir’s forecasting point from February 25th, 2021 to May 25th, 2050 hours of data in 2021 from February 25, 2021 to May 25, 2021, the rainfall and water level are shown as Fig 4(a). The fluctuation of water level in the first 1900 hours is gentle, and that in the last 150 hours is large, which can test the effectiveness of the method proposed in this paper. Qiaodongcun forecasting point has1000 hours from May 29, 2020 to July 9, 2020, the rainfall and water level are shown as Fig 4(b). The data in the first 850 hours basically changed little, but the peak water level in the next 150 hours changed rapidly, which can be used to detect the extreme changes of the water level targeted by this method. In general, 90% of the data of the water level and related disaster causing factors of the two forecasting points are used for training, and 10% of the data are used for verification. There are about 20 forecasting points around Qixi reservoir and Qiaodongcun forecasting point, which are applied to data reasoning and combination of LLM and KG. The data are normalized for statistical analysis.

From the change of actual rainfall and water level, the rainfall and water level of the two forecasting points are not completely corresponding, and there is a certain lag. The change of water level does not have periodic regularity, especially for the short-term 24 hours and medium-term 1-7 days of water level, accurate and real-time prediction has certain challenges.

### 4.2 Watershed-internal KG

**Fig 5:**
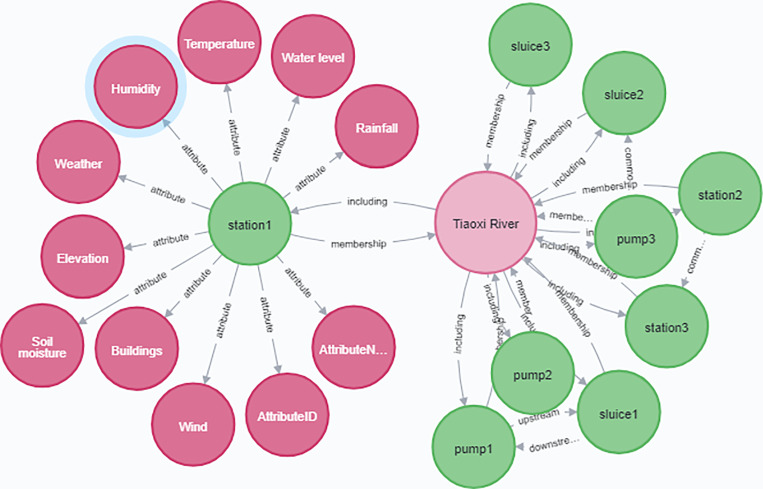
The WCE’s partial KG including watershed relationship and internal attribute.

The KG of water level forecasting was constructed, the WCE’s partial KG is shown in Fig 5. KG can organically organize hydrological, meteorological, geographic, and social longitudinal data together. Users click on forecasting points on the water conservancy map, and the system automatically retrieves information related to upstream and downstream tributaries, hydrology, meteorology, and geography through a small watershed KG. It constructs input factors for water level forecasting and calls DL forecasting methods such as RNN and LSTM to predict water levels at forecasting points in real time. In this experiment, the input factor data used as dynamic KG data can be obtained in real-time, supporting the data demand for large-scale water level forecasting points in watersheds. The KG can find data in a timely manner, facilitate data retrieval, and flexibly organize data. Optimization of storage structure: Adopting optimized storage can save storage space, improve access efficiency, and provide data security for real-time training of DL on data. The efficiency of storage has been improved, and the interpretability has become stronger.

Through data fusion, the extraction and alignment of water conservancy object entities, relationships, and attributes are achieved, and a dynamic water conservancy KG is constructed to correspond to real-time dynamic water conservancy data, achieving retrieval of water conservancy knowledge and dynamic data. Based on inference rules, conduct knowledge inference and mine small watershed knowledge and corresponding dynamic real-time data hidden in the KG of small watersheds. It combines with knowledge in the field of water conservancy, define inference rules, based on the existing KG of small watershed information. Table 5 shows some inference rules and data.

**Table 5:** Association point and relationship.

ID	Association point	Relationship	Data	Inference
1	Station 2	upstream	Water level, rain, weather, elevation, soil moisture, wind direction, wind speed, et al.	(River, membership, reservoir), (hydrological station, belonging to, reservoir) → (hydrological station, located in, river)
2	Station 2	downstream	Water level, land cover, et al.	(River, membership, reservoir), (hydrological station, belonging to, reservoir) → (hydrological station, located in, river)
3	Pump 1	Common water conservancy unit	Water level, rain, et al.	(pump station/sluice, including water intake), (pump station/gate station, belonging to, lake), (lake, belonging to, watershed partition) → (Water intake, belonging to, watershed partition)
4	Sluice 2	Same watershed	Water level, rain, et al.	(pump station/sluice, including water intake), (pump station/gate station, belonging to, lake), (lake, belonging to, watershed partition) → (Water intake, belonging to, watershed partition)

### 4.3 Mountain flood level forecasting

#### 4.3.1 Forecasting the experimental environment.

Python is a programming language that can provide RNNs models, and with PyTorch libraries are efficient tools for predictive data analysis. The extensible library to create LSTM layers on PyTorch at GitHub (https://github.com/) is used. Calculations were carried out with the CPU of intel(R) Xeon(R) E-2176M, double cores, 2.70GHz and 32 GB RAM.

The model’s traditional statistics measures, equations and meaning are as Table 6. Mean Square Error (MSE) is used to measure the difference between the predicted value and the real value. Mean Absolute Error (MAE) reflects real errors and is more sensitive to smaller errors. Nash-Sutcliffe Efficiency coefficient (NSE) measures the relative error between the predicted value and the real value, verifies the quality of hydrological model simulation results. The *i* is the sequence number of time series elements, the *m* is the number of elements, $y_{i}$ is the actual observation value of water level, and $\hat{y_{i}}$ is the predicted value of the prediction model on the variable.

**Table 6:** Model’s measure methods.

Name	Equation	Range	Impact and effect
Mean Square Error (MSE)	$MSE=\frac{1}{m}\overset{m}{\underset{i=1}{\sum }}{\left(y_{i}-\hat{y_{i}}\right)}^{2}$	[0, +∞)	When the predicted value completely matches the true value, it is 0, which is a perfect model; the larger the error, the greater the value. It affected by extreme values.
Mean Absolute Error (MAE)	$MAE=\frac{1}{m}\overset{m}{\underset{i=1}{\sum }}\left|y_{i}-\hat{y_{i}}\right|$	[0, +∞)	When the predicted value completely matches the true value, it is 0, which is a perfect model; the larger the error, the greater the value. It has better robustness against outliers.
Nash–Sutcliffe Efficiency coefficient (NSE)	$NSE=1-\frac{\sum_{i}{\left(y_{i}-\hat{y_{i}}\right)}^{2}}{\sum_{i}{\left(y_{i}-\bar{y_{i}}\right)}^{2}}$	(- ∞ ,1]	NSE = 1: it indicates that the simulated value of the model is completely consistent with the observed value. 0 ≤ NSE < 1: the model has certain prediction ability. NSE = 0: there is no difference between the prediction effect of the model and the average value. NSE < 0: it means that the prediction effect of the model is worse than the average value.

#### 4.3.2 Forecasting parameter configuration.

The forecasting models include LSTM, KG-LSTM and LLM-KG-LSTM. In order to verify the effectiveness of watershed internal KG and LLM, RNN and GRU, which are similar to LSTM, were used for comparison. The models have the main parameters’ value and meaning at Table 7. The parameters are set as the best result obtained through adaptive calculation based on the model. The parameters are also accidental and empirical. Through experimental tests, for example, the use of RELU in activation is obviously due to softmax, because RELU can prevent the gradient from disappearing in terms of its advantages in nonlinear prediction. Loss function is better trained with MSE, and it can converge effectively even with a fixed learning rate. The gradient of MSE loss increases with the increase of MSE loss, and decreases when MSE loss approaches 0. This makes the results using MSE model more accurate at the end of training.

**Table 7:** The models’ training parameters.

Parameter	Value	Meaning
Units	64	Positive integer, dimensionality of the output space.
Learning Rate	Adam (Adaptive Learning Rate)	The proportion of inputs that need to be discarded.
Activation	RELU	Activation function
kernel_regularizer	tf.keras.regularizers.l2()	Regularizer function applied to the kernel weights matrix.
Adam	0.0001	Adam optimization function and value.
Loss	MSE	Loss function
Metrics	accuracy	List of metrics to be evaluated by the model during training and testing.
Batch_size	8	Integer or None. Number of samples per batch of computation.
Epochs	500	Number of epochs to train the model.

Furthermore, it is important to note that the boundary conditions for modeling are maintained as consistently as possible to facilitate comparisons among different models. This involves keeping the input data, the number of layers, the number of neurons, the dropout rate, and the number of training epochs identical across models. Consequently, each model acquires a unique set of parameters while sharing the same structural framework, thereby reflecting information from various catchments that can be evaluated based on simulation outcomes. Once training is completed, the values are denormalized to enable comparison with observed data.

#### 4.3.3 Forecasting result.

**Fig 6:**
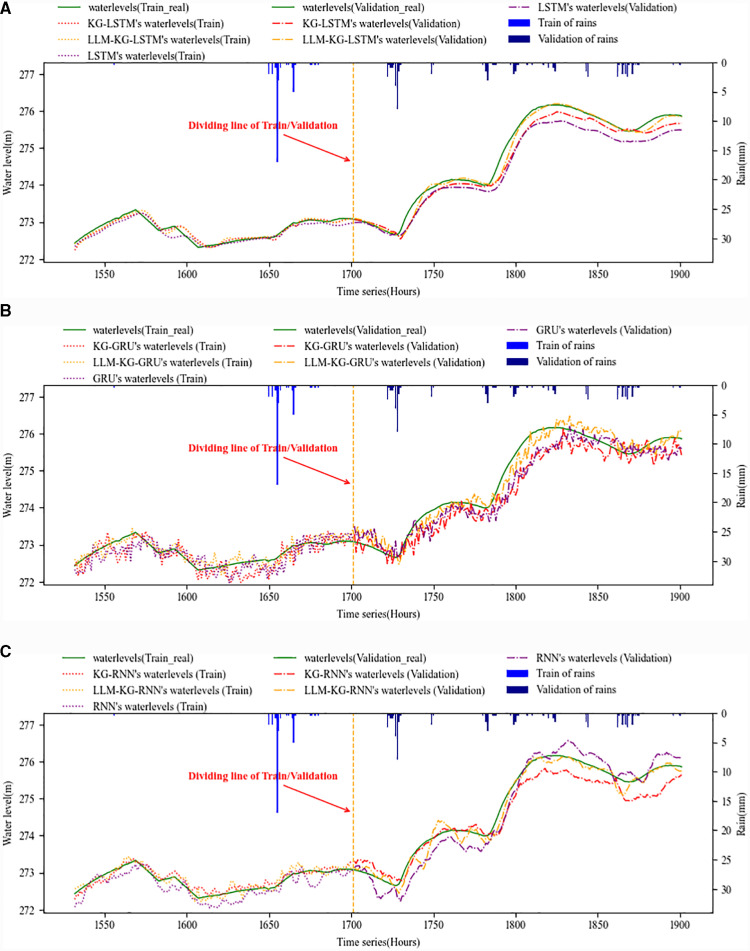
Forecasting method’s training and validation phase of Qixi Reservoir forecasting point. (a) **LSTM’s forecast curve by using watershed-internal KG and LLM**. (b) **RNN’s forecast curve by using watershed-internal KG and LLM.** (c) **GRU’s forecast curve by using watershed-internal KG and LLM.**

**Fig 7:**
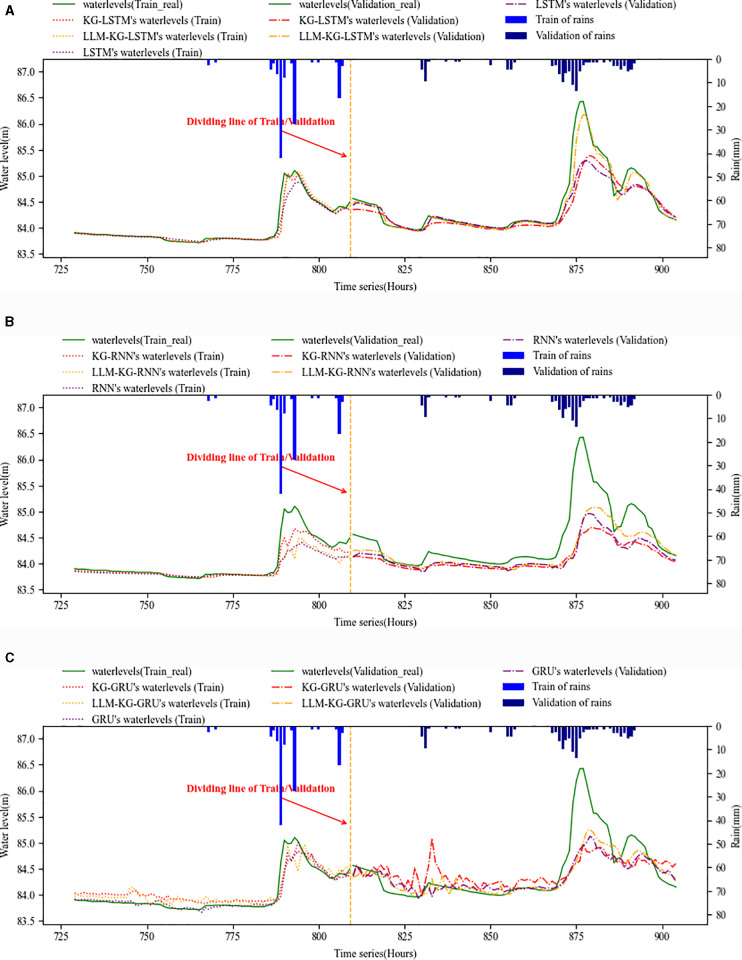
Forecasting method’s training and validation phase of Qiaodongcun forecasting point. (a) LSTM’s forecast curve by using watershed-internal KG and LLM. (b) RNN’s forecast curve by using watershed-internal KG and LLM. (c) GRU’s forecast curve by using watershed-internal KG and LLM.

The experiment includes the training and validation phase. Figs 6 and 7 show the training and validation effects of LSTM with watershed-internal KG and LLM-KG, and the respective methods are applied to Qixi and Qiaodongcun forecasting points respectively. The methods include LSTM, KG-LSTM and LLM-KG-LSTM. At the same time, we used RNN and GRU as the control method of KG and LLM. The figures include the water level and rain, the water level include the real data (green solid line) and the forecasting data, the forecasting data include the DL methods (RNN, GRU and LSTM, purple line), KG (red line) and KG-LLM (yellow line).

From a qualitative point of view, the prediction curve of LSTM in Figs 6 and 7 is smooth and stable, with clear trend, which is in line with the basic characteristics of LSTM. In addition, after using watershed-internal KG and LLM, the prediction curve is closer to the actual water level curve, whether LSTM or RNN and GRU. At Qixi forecasting point, the overall performance of LSTM prediction is better than RNN and GRU when the water level changes gently, but KG and LLM can effectively promote the accuracy of LSTM prediction regardless of which algorithm. At Qiaodongcun forecasting point, KG and LLM have played a key role in improving the water level under the condition of rapid change. In particular, adding KG and LLM at the same time makes the prediction curve at the time of verification closest to the actual value, which also shows that the prediction is stable and the internal data organization is flexible.

Table 8 shows the quantitative result data of MSE, MAE, NSE and calculate time consumption. All data are normalized results for comparative analysis.

**Table 8:** Comparison of key indicators for model forecasting.

Method	Training				Validation			
	MSE	MAE	NSE	Time (s)	MSE	MAE	NSE	Time (s)
**(a) Forecasting result of Qixi Reservoir about MSE, MAE, NSE and time consumption**								
LSTM	0.1485	0.1662	0.8724	209.86	0.1514	0.1784	0.8633	0.11
KG-LSTM	0.1368	0.1519	0.9131	224.11	0.1361	0.1659	0.9121	0.18
LLM-KG-LSTM	0.1169	0.1427	0.9321	246.60	0.1196	0.1444	0.9312	0.20
RNN	0.2113	0.2538	0.8932	192.37	0.2231	0.2609	0.8419	0.11
KG-RNN	0.1931	0.2219	0.9013	224.58	0.2149	0.2395	0.8821	0.19
LLM-KG-RNN	0.1636	0.1953	0.9348	261.37	0.1749	0.2001	0.8991	0.26
GRU	0.1942	0.2314	0.8123	112.31	0.2123	0.2765	0.8873	0.12
KG-GRU	0.1623	0.2045	0.8712	142.17	0.1945	0.2359	0.9002	0.14
LLM-KG-GRU	0.1413	0.1658	0.9013	195.24	0.1501	0.1581	0.9015	0.25
**(b) Forecasting result of Qiaodongcun about MSE, MAE, NSE and time consumption.**								
LSTM	0.1505	0.1699	0.8624	194.85	0.1594	0.1821	0.8613	0.11
KG-LSTM	0.1451	0.1659	0.9001	203.13	0.1472	0.1795	0.8928	0.16
LLM-KG-LSTM	0.1219	0.1421	0.9219	209.27	0.1291	0.1461	0.9217	0.21
RNN	0.2113	0.2538	0.8932	192.37	0.2231	0.2609	0.8419	0.11
KG-RNN	0.1931	0.2219	0.9013	224.58	0.2149	0.2395	0.8821	0.19
LLM-KG-RNN	0.1636	0.1953	0.9348	261.37	0.1749	0.2001	0.8991	0.26
GRU	0.1942	0.2314	0.8123	112.31	0.2123	0.2765	0.8873	0.12
KG-GRU	0.1623	0.2045	0.8712	142.17	0.1945	0.2359	0.9002	0.14

From the perspective of MSE, it can reflect the prediction error. At Qixi reservoir forecasting point, in the training phase, watershed internal KG reduced the MSE of LSTM by 1.17%, and LLM and KG further reduced the MSE by 2.0%, that is, a total of 3.17%; In the validation phase, KG reduced the MSE of LSTM by 1.53%, and LLM and KG further reduced the MSE by 1.65%, that is, a total of 3.18%. At Qiaodongcun forecasting point, in the training phase, KG reduced the MSE of LSTM by 0.51%, and LLM and KG further reduced the MSE by 2.31%, that is, a total of 2.83%; In the validation phase, KG reduced the MSE of LSTM by 1.22%, and LLM and KG further reduced the MSE by 1.81%, that is, a total of 3.03%. Overall, KG and LLM improved the accuracy by about 3%.

MAE reflects the real error and is more sensitive to smaller errors. At Qixi reservoir forecasting point, LLM and KG reduced the MSE of LSTM method by 2.35% in the training phase; in the validation phase, LLM and KG further reduced MSE by 3.40%. At Qiaodongcun forecasting point, LLM and KG reduced MSE by 2.78% during the training phase; in the validation phase, LLM and KG further reduced MSE by 3.40%. Overall, KG and LLM improved the accuracy by about 3%, which was consistent with the results of MSE.

NSE reflects the prediction performance of the model. The closer to 1, the better the comprehensive performance of the model. From the verification stage, at Qixi reservoir forecasting point, the NSE of LSTM increased from 0.8633 to 0.9312, and the total increase of LLM-KG-LSTM is 6.79%. At Qiaodongcun forecasting point with extreme changes, the NSE of LSTM increased from 0.8613 to 0.9217, and the total increase of LLM-KG-LSTM is 6.04%. Obviously, the application of LLM and KG has effectively improved the performance of LSTM prediction model.

From the perspective of time consumption, taking Qixi reservoir forecasting point as an example, the training time of LSTM increased by 35s, that is, LLM-KG-LSTM increased by 16.74% compared with LSTM, LLM and KG do not significantly increase the training burden, far less than 1 hour, and the real-time performance of the model do not change under the frequency of data acquisition, update and calculation every hour.

From the control test, no matter RNN or GRU, when KG and LLM are used, the MSE, MAE and NSE of the validation results will be significantly improved, which shows the universality of KG and LLM processing data for DL type prediction method. However, the overall performance of LSTM is not as good as that of LSTM, which shows that LSTM has better prediction effect under the full mining of data information.

In general, MSE, MAE and NSE all reflect that they are effective for water level prediction, especially the increased KG and LLM have obvious improvement effect, the time consumption is acceptable. The forecasting effect of disaster causing factors based on KG reasoning is good, and KG-LSTM and LLM- KG-LSTM are significantly better than the basic LSTM. KG and LLM are used to effectively improve the flexibility of disaster causing factors and improve the accuracy of water level forecasting.

## 5 Discussion

### 5.1 Watershed-internal knowledge graph

The rapid development of water conservancy informatization has achieved significant results in the prevention of mountain flood disasters, and the overall level of intelligent forecasting in water conservancy has been improved. However, the shortcomings and weaknesses in emergency disaster management have also been exposed [45], mainly divided into the following points.

(1) There is no unified standard for the data integration. Currently, water conservancy perception equipment and facilities are diverse, communication protocols are different, and there is no unified interface specification, resulting in difficulties in data integration technology, a large amount of access workload, and difficulty in online ubiquitous diagnosis of equipment [46].
(2) There is no perfect solution for integrating heterogeneous data from multiple sources, and there are heterogeneity, distribution, and autonomy among data sources. The data types include both structured data such as digital and relational data, as well as unstructured data such as images and audio. Due to the lack of solutions for multi-source heterogeneous data warehousing in the industry, it is currently difficult to integrate and share heterogeneous data [47].

The watershed-internal KG of water conservancy has good logical organization and can provide good guidance for the inference and storage of water conservancy data, the organization of watershed-internal KG has high advantages in both data acquisition and information organization, surpassing traditional information models. Through data processing, water conservancy BD can be stored in relational databases.

Based on watershed-internal KG for small watershed data processing, data fusion is used to extract and align entities, relationships, and attributes of water conservancy objects, construct a dynamic water conservancy KG, correspond to real-time dynamic water conservancy data, and achieve retrieval of water conservancy knowledge and dynamic data. Based on inference rules, conduct knowledge inference and mine small watershed knowledge and corresponding dynamic real-time data hidden in the KG of small watersheds.

The comparison of watershed-internal KG with traditional knowledge models and KG is shown in Table 9. By constructing a dynamic knowledge spectrum for the semantic knowledge model of water conservancy objects, the improved knowledge model has been improved in terms of model architecture, semantic structure, technical implementation, and support functions compared to fragmented information points and simple knowledge models, enhancing the completeness of knowledge. The watershed-internal KG formed through standardized modeling has richer semantics and is easy to implement. It is particularly noteworthy that dynamic KG outperform traditional KG in terms of semantic knowledge elements of knowledge nodes, modeling efficiency, and dynamic information retrieval.

**Table 9:** Characteristics comparison of structure of forecasting.

Model evaluation factor	Only LSTM	KG-LSTM	LLM-KG-LSTM
Organization of data	None	Strict organizational structure	Flexible and strict
Dynamics of data	Low	Middle	High
Interpretability	None	Based on the distribution structure of impact factors	Based on principle
Accuracy of forecasting	Middle	High	High

In addition, the watershed data itself also poses a certain challenge to the generation of KG. The Qiantang River Basin is known as the “Zhijiang River”. The tributaries in the mountainous areas are complicated, the data of the tributaries are large, and the relationship network of forecasting points is complex. The selection of Qiantang River Basin has a good representation in terms of complex data.

### 5.2 Data’s process of knowledge graph

The quality of water conservancy data directly determines the effectiveness of intelligent water conservancy applications. In the early phase of feature engineering in artificial intelligence, data processing and storage are required. However, there is less research on how to scientifically obtain, organize, and store relevant data, and the quality of data directly concerns the accuracy of forecasting. The organization of large-scale data and attention to the reliability of continuous forecasting.

The major problem with KG is the slow updating of knowledge. Especially, KG established only has organizational structure knowledge and lacks real-time data support. As a result, there is only knowledge reasoning and lack of real-time data. For example, knowing the upstream and downstream relationship between a certain forecasting point and other mountain torrent forecasting points, but lacking their own real-time water level relationship, is not enough for real-time water level forecasting, which requires large-scale real-time data to support KG. Combining the KG and LLM, taking into account the knowledge structure and real-time data can better solve the problem of mountain torrent water level forecasting. In addition, the cleaning and storage of real-time data based on KG can improve the utilization rate of data. Real time data needs the organization of KG in every link of data identification, information interaction, data cleaning and storage.

The real-time nature of the data determines the accuracy of the forecasting. Most papers consider the accuracy of using historical data for training, while how to use real-time data for practical forecasting is less discussed and cannot be introduced from theory to practice. The acquisition and organization of real-time data is very important. How to get the real-time data and send it to the forecasting model for training and forecasting in time, then the organization ability of the whole data is taken part in the postgraduate entrance examination, KG is very useful. In addition, it also needs the continuous data training ability of the forecasting model and the error correction of the forecasting model by using real-time monitoring data to reduce the continuous spread of error. In addition, there is a certain error in the acquisition of data, which requires mutual verification of multi-path and multi-modal data to improve the reliability of data.

Specifically, the role of watershed-internal KG in BD analysis mainly includes the following aspects.

(1) Data integration and management. Watershed-internal KG can integrate and manage data from different sources, making it easier to analyze and query data. Meanwhile, the semantic associations between WCEs and relationships in the KG can also help us understand and analyze data more accurately.
(2) Data mining and analysis. Watershed-internal KG can provide more accurate and comprehensive data support for data mining and analysis. By analyzing entities, attributes, and relationships in a watershed-internal KG, potential associations and patterns can be discovered, helping us better understand and predict trends and changes in data.
(3) Human computer interaction and decision support. Watershed-internal KG can help people understand and analyze data more intuitively through visualization and interaction. Meanwhile, intelligent decision support systems based on KG can also help people make more accurate and comprehensive decisions.

However, there are also some challenges, especially the lack of data, the lack of WCE at forecasting points, and the unclear relationship between WCE within and between watersheds in small mountain watersheds. In addition, the disaster causing data is lack of consistency check and difference, resulting in the data cannot be applied in real time. The KG proposed in this paper supplements the proximity value of the forecasting point with the relevant data through KG reasoning. It is worth noting that with the increase of sensing equipment, the damage of sensing equipment in harsh environment can be quickly supplemented. Of course, too much may increase the complexity of the system.

### 5.3 LLM for water conservancy

Based on the knowledge mining technology of multi-source water conservancy data based on LLM, the application of water conservancy professional computing and intelligent decision-making can realize the ability of interactive docking of water conservancy objects and professional models in multi-business fields. The ontology construction method of multi-service fusion KG is to connect the LLM with the content of water conservancy knowledge base such as business rules, subject knowledge, expert experience, historical scenes and human natural language understanding ability, so as to realize the business application scenario driven water conservancy LLM to carry out water conservancy professional calculation and intelligent decision-making. The physical process and expected impact of different scheduling schemes are deduced through LLM driven water conservancy professional calculation, and intelligent decision-making assistance based on LLM interaction and water conservancy knowledge constraints is realized, so as to enhance the efficient, scientific and intelligent level of water conservancy business decision-making.

The clutter of disaster causing data leads to the infeasibility of forecasting. KG-LLM effectively combines water conservancy data. Obtain the diversity of routes: through the Internet, databases, real-time IoT, etc., we can combine KG to find more valuable water level change related information, improve the reliability of data and the flexibility of forecasting data sources. The preparation time for flood forecasting has become shorter and can be carried out in real time. The experimental speed has increased, and the problem can be better solved with sufficient and reliable data sources.

### 5.4 Forecasting model’s performance

Compared with the basic RNN and GRU, LSTM has high accuracy and reliability. As can be seen from Figs 6 and 7, the overall stability of the LSTM series, RNN and GRU have a certain vibration, while the LSTM is relatively smooth, with a clear trend and no overfitting but with a certain time lag, indicating that the data collection affecting water level elements has a certain time lag, while KG and LLM can find more relevant influencing factors, improve the problem of time lag and improve the accuracy of prediction.

In addition, in terms of data selection, the complexity of the Qixi reservoir and Qiaodongcun data sets in Zhejiang Province can fully reflect the performance of the model. Of course, due to the small number of mountain torrents with complete data sets, the water conservancy data set is not open, or the time complexity is simpler than ours. We will process more complete data sets through multimodal methods, especially when there are many data transients caused by extreme weather, to verify the universal adaptability of the model.

Accuracy and resource consumption have some contradictions with real-time performance. LSTM model takes a long time. In addition, LLM and KG further extend the data processing time, and KG based on LLM needs to be updated immediately, otherwise it will lead to data lag. Therefore, we can transform the data structure, limit the KG range, and realize the LLM remote call mode, which will be the premise for us to carry out large-scale prediction of small watersheds, and also our future improvement direction.

## 6 Conclusions

This study investigated the data processing method of real-time acquisition, organizing and storage for real-time mountain flood forecasting, The main conclusions are as follows.

(1) The advantage of using watershed-internal KG and LLM to organize data is that it can organically organize hydrological, meteorological, geographic, and social longitudinal data together, and conduct inferential mining of disaster causing factors, which is more reliable than blindly identifying the data types input by the model. At the same time, watershed-internal KG have optimized effects in the database storage design of key elements for water level forecasting.
(2) The stability of real-time water level forecasting based on LSTM is good, the accuracy of the LLM-KG-LSTM model is enhanced by 3% compared to the standard LSTM model, and it can meet the continuous forecasting frequency during flood season, with one forecasting frequency per hour.
(3) The method of flood forecasting is changed from the perspective of paying attention to the forecasting algorithm to the perspective of multi-dimensional disaster data processing and analysis and algorithm parallel. In addition, KG and LLM are good choices for large-scale forecasting points in small watersheds, complicated water networks such as river interweaving, mutual influence of forecasting points, and insufficient data at single forecasting point. Our method will have advantages in small watershed large-scale forecasting points.

In our future work, we further deepen LLM, build LLM based on hydrology, and build a knowledge platform for hydrological forecasting, and further study the effective principle of KG, such as upstream water levels and precipitation. And in the future, the effectiveness of intense water level changes will be verified in different types of waters.
